# Patient-centered medicine and patient-oriented research: improving health outcomes for individual patients

**DOI:** 10.1186/1472-6947-13-6

**Published:** 2013-01-08

**Authors:** José A Sacristán

**Affiliations:** 1 Clinical Research Department, Lilly Spain, Avda. de la Industria, 30, Alcobendas, Madrid 28108, Spain

**Keywords:** Patient-centered care, Research, Preferences, Patient-reported outcomes, Heterogeneity, Clinical trials

## Abstract

**Background:**

Patient-centered medicine is developing alongside the concepts of personalized medicine and tailored therapeutics. The main objective of patient-centered medicine is to improve health outcomes of individual patients in everyday clinical practice, taking into account the patient’s objectives, preferences, values as well as the available economic resources.

**Discussion:**

Patient-centered medicine implies a paradigm shift in the relationship between doctors and patients, but also requires the development of patient-oriented research. Patient-oriented research should not be based on the evaluation of medical interventions in the average patient, but on the identification of the best intervention for every individual patient, the study of heterogeneity and the assignment of greater value to observations and exceptions. The development of information-based technologies can help to close the gap between clinical research and clinical practice, a fundamental step for any advance in this field.

**Summary:**

Evidence-based medicine and patient centered medicine are not contradictory but complementary movements. It is not possible to practice patient-centered medicine that is not based on evidence, nor is it possible to practice evidence-based medicine at a distance from the individual patient.

## Background

Patients are playing an ever-greater role in the healthcare systems of developed countries. The healthcare systems in these countries are evolving from a doctor–patient relationship based on paternalism and the authority of the doctor to a model based on the ethical principle of patients’ autonomy, where patients are transforming into customers, have more and better information, and want to take a more active role in making decisions that affect them [1]. These changes are reflected by the principal of “*nothing about me without me*” and the development of patient-centered medicine (PCM) [2].

Patients have always been the focus of medical care, which means the concept of PCM seems to be redundant and provocative. However, emphasizing the existence of a healthcare process that is focused on the patient implies explicit recognition that other ways of practicing medicine exist. In fact, in the last few decades, healthcare has been characterized by a provider-centered model [2] with an emphasis on the evaluation and treatment of diseases rather than the evaluation and treatment of patients. These and other characteristics of PCM, compared with those of traditional clinical medicine, are summarized in Table 1.

**Table 1:** Characteristics of the traditional medical model and patient-centered medicine

**Traditional medical model**	**Patient-centered medicine**
Provider-centered model	Patient-centered model
Founded on the principles of beneficence and authoritarianism	Founded on the principle of autonomy
Disease-oriented care	Patient-oriented care
Focuses on outcomes of importance for physicians and regulators	Focuses on outcomes of importance for patients
The patient’s perspective is usually ignored	The patient’s preferences, objectives and values are taken into account during decision making and delivery of healthcare
Compliance with the physician’s decisions	The patient and physician share decision making
Improve outcomes for the average patient	Improve outcomes for the individual patient
Population-oriented research	Patient-oriented research

PCM is developing alongside the concepts of personalized medicine and tailored therapeutics [3]. Advances in pharmacogenomics and personalized medicine, the objective of which is to provide tailored therapeutic approaches, can be summed up with the catch-phrase “*one size does not fit all*”. However, the concept of PCM is broader than personalized medicine, as far as the term is usually understood. Personalized medicine reaches it full significance as the opposite of “depersonalized medicine” or “illness-oriented care”, the aim of which is to treat the illness, not the patients with the illness [4]. The practice of PCM requires the dedication of greater effort to improve our knowledge of the psychological, social and cultural aspects that may affect disease prognosis in individual patients.

PCM can be defined as the medical practice aimed at improving the health outcomes of individual patients in everyday clinical practice, taking into account their preferences, objectives and values, as well as the available economic resources. PCM requires a change in the relationship between the doctor and the patient, and also has implications on medical research, legislation, education, and medical ethics. This work discusses the objectives and characteristics of PCM, and the implications of this model for medical research and clinical practice.

## Discussion

### Objectives of PCM

If the objective of PCM is to improve the health outcomes for individual patients, then we need to define the outcomes that should be measured, and the significance of improving these outcomes. In research to date, the outcome measures are usually those that are most important to the doctors and regulators. Through PCM, the focus should shift to outcomes that are important to the patients themselves [5]. For example, in the context of diabetes, only 18% of the randomized clinical trials (RCTs) evaluated included outcomes that are important to patients, such as amputations, stroke, loss of vision, and other outcomes that affect quality of life [6]. Similarly, in cardiovascular disease, only 16% of studies included at least one instrument used to measure patient-reported outcomes (PRO) [7].

Clearly, it is necessary to measure final outcomes, not just processes such as compliance to good practice guidelines achieved by the doctors [8]. Although “hard” outcomes such as mortality or serious complications should be measured, “soft” outcomes such as symptoms, physical functional status, social and role functions, or quality of life—“goal-oriented patient care outcomes”—better reflect the patients’ perspective [9]. Patient-centered care means attending to the physiological and social aspects of an illness that cannot be reduced to a single objective measure. Sometimes, evidence on patient preferences might only be captured with qualitative research, not from questionnaires or blood parameters [10].

The regulatory authorities are now promoting the use of PRO, and both the EMA and the FDA have published guidelines to include these measures in RCTs, so that the labels of the medicines can include this information [11,12]. Many scales designed to measure PRO were published in recent years, although these publications were not always accompanied by appropriate validation. For example, in the areas of diabetes and schizophrenia, only 44% [13] and 35% [14], respectively, of the variables have been adequately validated.

Referring to what signifies a relevant improvement in outcomes, we need to remember that “*a difference, to be a difference, must make a difference*.” A statistically significant difference does not necessarily represent a clinically relevant difference. Clinical relevance is related to the concept of clinical usefulness, “a multifactorial parameter that describes the relevance and end usefulness of an intervention in patient care” [15]. From the point of view of PCM, clinical usefulness should consider the improvements in outcomes that are important to the patients (i.e., survival, quality of life and functional ability), and establish the threshold and the lowest level of change considered relevant by the patients [16,17]. Clinical usefulness should be conceptualized to include the dimension of “personal utility” for individual patients [15].

An essential element of PCM is the active participation of patients in the decision-making process [18]. The concept of shared decision-making necessitates a profound change in the traditional model of the doctor–patient relationship [19]. This new model is based on greater information exchange. In particular, the doctor must provide the patient with full and comprehensible information on the disease, prognosis, and the potential benefits and risks of the different therapeutic options available. In turn, the patient must provide the doctor with preferences and attitudes towards the disease and the risks involved [20]. A well-informed patient is essential to ensure the decision-making can be based on the patient’s preferences. For the doctor, the fundamental change is not in the individualization of treatments, but in the individualization of therapeutic decisions, where the patient’s preferences play a fundamental role.

Patients and doctors need to be educated in this new paradigm. The role of the healthcare professional should change “from experts who care for patients to enablers who support patients to make decisions” [20]. The doctor should be able to adapt the information available to suit the requirements of individual patients, so that the patient, after giving informed consent, can establish personal trade-offs for the available treatments [21]. Factors such as level of adherence to medication, degree of tolerance to an adverse effect, past experience, and health objectives can have a decisive influence on the patient’s preferences.

In many occasions, there is one clearly superior path, in which case the patient’s preferences may play little or no role. Generally, patients evaluate prevention and increased life expectancy positively [21]. However, in many medical decisions, there is more than one reasonable option [19]. For example, a patient may prefer a more economical treatment even if it is less effective, a more conservative surgery because of greater emphasis on quality of life than on life expectancy, or may refuse chemotherapy because the adverse effects are considered unlikely to compensate for the increased survival time [21]. Fortunately, obtaining information about a patient’s preferences does not require the use of complex scales or sophisticated statistical tests. A good anamnesis, and asking the patient what he prefers, is an excellent strategy for personalized research. If personalized medicine is determined by the patient’s preferences [22], it seems reasonable that this information should be included in their clinical histories [1].

For the patients, decision aids have been developed that could help them in this process [23], but the patients would need to fundamentally change their attitudes and have a greater level of literacy to fully benefit from these aids. Patients must clearly describe their expectations and choices, and accept the uncertainty that surrounds each medical decision. The patients must also understand that their responsibility is to not just follow their doctors’ orders but also take joint responsibility for the decisions and therapeutic strategies agreed with their doctors, and accept the results of these decisions. Providing patients with greater empowerment over medical decisions involves a switch from the current situation of unconscious certainty to a situation of conscious uncertainty.

PCM can only be achieved through the development of patient-oriented research, the results of which could be translated into clinical decisions that increase the effectiveness of medical care. Both elements will be analyzed in the following sections.

### PCM and patient-oriented research

One of the fundamental characteristics of medicine in the second half of the 20^th^ century has been its focus on epidemiological and social factors, in an attempt to maximize the health results in the general population. This population-oriented focus is intimately linked with the development of clinical epidemiology and evidence-based medicine (EBM) [24]. This approach has formed the paradigm of scientific medical research, placing RCTs as the gold standard for evaluation of healthcare interventions, contributing to the standardization of healthcare and the development of evidence-based guidelines.

The development of PCM necessitates closing the gap between clinical research based on population studies and clinical practice, with a shift to human-centric factors and ultimately to improve the health outcomes for individual patients [25]. An important objective of patient-oriented research should not be to predict what percentage of patients will respond to a particular intervention, but rather to identify which patients are likely to respond. Table 2 compares the main features of population-oriented research, typical of EBM, and patient-oriented research.

**Table 2:** Characteristics of population- and patient-oriented research

**Population-oriented research**	**Patient-oriented research**
Paradigm: Randomized clinical trials	No paradigm: observational and experimental research
Focuses on the “generalization” of results	Focuses on the “individualization” of results
Efficacy in average patients	Effectiveness in subgroups of patients and individual patients
Absolute efficacy	Comparative effectiveness
Identify the percentage of patients who will respond to an intervention	Identify which options are more effective for which patients
Evaluation of interventions	Evaluation of patients and their diseases
Analysis of homogeneity	Analysis of heterogeneity
*A posteriori* subgroup analysis	Subgroups identified *a priori*
Aggregation: the study of commonalities	Disaggregation: the study of differences
Inductive logic	Hypothetic-deductive logic
Exploratory observations and confirmatory trials	Exploratory trials and confirmatory observations
Minimizes the value of observations, exceptions, and case series	Assigns greater value to observations, exceptions, and case series
Distinction between clinical practice and research	Integration of clinical practice and research
From bench-to-bedside	From bedside-to-bench
Evidence-based medicine	Medicine-based evidence

The development of PCM has coincided with that of comparative effectiveness research (CER) [26]. The joint development of PCM and CER is not accidental. CER has been defined as “the generation and synthesis of evidence that compares the benefits and harms of alternative methods to prevent, diagnose, treat, and monitor a clinical condition or to improve the delivery of care. The purpose of CER is to assist consumers, clinicians, purchasers, and policy makers to make informed decisions that will improve healthcare at both the individual and the population levels” [27].

CER and PCM share a common objective: to identify which options are more effective for which patients [28]. For this reason, CER could become an excellent platform from which to develop a patient-oriented research strategy that could serve as a basis for PCM. To achieve this, it will be necessary to change the beliefs that are strongly entrenched in the scientific community. Predictably, in a research environment that is strongly influenced by EBM, large pragmatic RCTs are considered the “logical” starting point for CER [29]. These studies compare clinically relevant options, enroll a diverse clinical population recruited from a variety of practice settings, and measure a broad range of relevant health outcomes [30]. Observational studies using patient records and other databases have also been promoted, particularly in settings where RCTs are not feasible [31].

The objective of these large pragmatic studies is to reach conclusions that are generalizable to all types of patients with a specific disease. From the context of traditional clinical research, this is a reasonable objective, as one of the main limitations of RCTs is the generalizability of their results [32]. However, from the context of patient-oriented research, the main limitation of RCTs is not their lack of generalizability, rather the lack of individualization. Therefore, increasing the heterogeneity of the studies, by enrolling many different types of patients, is not the best solution as large RCTs and megatrials do not provide an adequate solution to this limitation, but actually makes it worse, meaning the “forest does not let us see the trees” [4].

Patient-oriented research requires an analysis of the heterogeneity of different kinds of patients [33]. The best decisions for the average patient are not necessarily the best decisions for an individual patient [34-37]. There are often subgroups of patients with different basal risk levels who respond better than the average patient to the same treatment, while other patients are not helped or may even be harmed by this treatment [38]. This heterogeneity of response may be “quantitative”, where one of the interventions is superior to another in all the subgroups or “qualitative” when one of the interventions is superior in some subgroups and worse in others [39]. In both of these situations, the strategy of expanding the inclusion criteria in RCTs and evaluating the effects of interventions in heterogeneous groups of patients actually dilute the observed effects, requiring larger sample sizes to detect statistically significant differences.

Some authors have reported that the future of clinical research should lie in the implementation of large RCTs designed to show small differences among heterogeneous groups of patients [40,41]. However, are large sample sizes necessary because the differences in efficacy of the treatments being compared are small, or are the differences small because the samples are too large and heterogeneous? If this is the case, perhaps we should consider smaller studies of homogenous subgroups defined *a priori* to detect clinically important differences.

Subgroup analysis involves improvements to patient classification systems and the development of phenotypic and genotypic markers. Additionally, it will be important to encourage studies focusing on the physiopathological mechanisms of diseases, factors that may determine differences in prognosis and response between subtypes of patients. It is equally important to fully evaluate the biological differences between patients and understand the psychological, social, and cultural differences that could modify personal preferences, as these could influence the patient’s preference for a specific alternative treatment, and condition the patient’s prognosis and final health outcomes.

*A priori* subgroup analysis represents an advance over analyses of the average patient, but one of its limitations is that classification is carried out on a single variable, such as sex, age, or the presence of a particular clinical characteristic. Unfortunately, these single variable analyses do not include all relevant patient information. For this reason, it is desirable to develop methods that would provide more complete information on all of the relevant characteristics of all patients, which would facilitate the jump from subgroup analyses to individual analyses. Several interesting methods, based on risk models, have been proposed to predict the effects of a specific treatment in individual patients [42].

The development of patient-oriented research requires a change in the type of reasoning. If the path from an individual patient towards the average patient requires data aggregation, the path back to the individual should follow the inverse process: disaggregation of data, the analysis of subgroups and individuals [43]. Although many patients are similar, all patients are different. Similarities between patients explain why inductive reasoning, under which generalizations are drawn from an accumulation of cases, has taken such a strong hold on research. A patient-oriented research perspective demands a shift towards hypothetic–deductive logic, in which RCTs primarily serve to generate a hypothesis (i.e., that one intervention is superior to another in some groups of patients), while observations would test the validity of the hypothesis for individual patients. Consequently, RCTs would be considered as “exploratory” and observations as “confirmatory” [43]. This change in thinking supposes that regulatory approval should no longer be regarded as the final step in hypothesis-testing, but as the hypothesis-generating step. Currently, most clinical studies are concentrated in the preauthorization phase and are oriented towards hypothesis generation. Predictably, in the future, a greater emphasis will be given to confirmatory investigation, carried out after regulatory approval.

Consistent with this approach, patient-oriented research should give much greater weight to clinical observations. It is important to note the role played by careful observation in the history of clinical studies of new drugs, such as that described by Roland Kuhn on the discovery of the antidepressant imipramine: “I have never used ‘controlled double blind studies’ with ‘placebos’, ‘standardized rating scales’ or the statistical treatment of records of large numbers of patients. Instead I examined each patient individually every day, often on several occasions, and questioned him or her again and again. Many of the patients were also under the observation of my assistants and nursing staff, and I always regarded their proposals and criticisms seriously” [44].

Case reports and case series may provide the weakest level of evidence, but are often the “first line of evidence” [45,46]. Individual cases are most useful when they show us the unexpected and, in research, the unexpected can signal a new truth [47]. But, if isolated observations are important, then so is the systematization of observations. Some authors have proposed prospective "formal case studies” to collect pure cases in which to test *a priori* hypotheses [48]. In a prospective case study, the investigator consciously and explicitly reflects on the theory, and uses it to develop a specific hypothesis or model that is subsequently tested in patients deliberately chosen to either confirm or reject the hypothesis [49].

### How can we improve the effectiveness of interventions in clinical practice?

One of the primary objectives of PCM is that the patients should derive greater benefit from the results of clinical research. In recent years, the concept of translational research has received significant attention. Translational research is defined as “the effective translation of new knowledge, mechanisms, and techniques generated by advances in basic science research into new approaches for the prevention, diagnosis, and treatment of disease” [50]. The expression “from bench-to-bedside” accurately reflects the objective of translational research, a type of research frequently characterized by the lack of communication between basic research and clinical research. This concept has also led to a true “death valley” [51], where basic research findings are rarely transformed into solutions for patients.

One way of increasing the effectiveness of biomedical research is to change the direction of research, in which we start with identifying the true requirements of the patients, the medical problems that still have no answers, or the exceptions that surprise clinicians. It is these questions arising in the clinics that should start the path towards basic research, in a direction *“*from bedside-to-bench”.

The term “translational research” also refers to “translation of the results from clinical studies into everyday clinical practice and health decision-making” [50]. Closing the gap between efficacy and effectiveness is fundamental to ensuring that the results of RCTs are translated into tangible improvements in patient outcomes. It has been claimed that patients could benefit more, and more patients could benefit, if the health services produce better results when delivering existing treatments than by introducing new treatments [52].

One of the reasons that the advances generated by EBM are not put into practice, or are done with considerable delays, is the huge gap between research and clinical practice [53]. Closing the gap between experiments and real life means eliminating the barriers between research and clinical practice. Clinicians should become more engaged in research and be provided with better methodological training. Perhaps more importantly, however, the research should be moved closer to clinics, become simpler, and be better incorporated into the daily activities of clinicians.

It is essential to eliminate the differences between doctors who carry out research and those who do not, between patients who participate in clinical trials and those who do not, and between the case report form of RCTs and the medical record [4]. Every medical act has the structure of an experiment, and every experiment starts with a patient [54]. The results of RCTs can take decades to transfer from the pages of a peer-reviewed medical journal to routine clinical practice, but anecdotes of adverse findings can transform clinical practice in an instant [55].

New information-based technologies, such as electronic medical records (EMR), can help to unite clinical research and medical care [56]. Researchers can exploit EMRs containing a vast amount of information generated by each medical interaction. The idea of applying randomization to EMRs would enable “randomized database studies”, which combine the advantages of observational studies and experimental studies [57]. The incorporation of decision aids could encourage quicker application of the knowledge acquired during research [58].

To date, EMRs have been used to aggregate data and to carry out large analytical observational studies of the average effectiveness of health interventions. From a patient-centered perspective, EMRs are potentially most useful to disaggregate data and to identify possible differences among patients. EMRs can be used to (1) assess how and in which particular patients interventions are applied in clinical practice; (2) analyze different response patterns, identify patient subgroups, and classify them in accordance with their risk factors and comorbidities; (3) help systematize the exceptions to treatment and the factors responsible for these exceptions; (4) understand a given intervention’s risk–benefit ratio at the individual level; (5) record patient preference and perception; and (6) support individual decisions by providing information about each patient. Integration of prediction models, risk calculators, and decision aids in EMRs may facilitate decision-making at the individual level, taking into account the benefits and risks anticipated for each patient [42,59]. Predicting the effects of a treatment for individual patients may enable doctors to practice individualized medicine in an evidence-based manner [42], while considering that the best test for a clinical prediction rule is not accuracy but rather improved clinical outcomes [60].

Closing the gap between efficacy and effectiveness also requires us to carry out strategies and programs that will help make potentially effective treatments truly effective [61]. It it of little use to develop more effective treatments if the patients do not use them correctly. We must stop focusing on measuring noncompliance with treatment, and instead endeavor to improve compliance. Patient-support programs have recently been developed, and are defined as “a service for direct patient–carer interaction/engagement designed to help management of medication and/or disease outcomes (e.g., adherence, awareness and education), or to provide healthcare professionals with support to their patients” [62]. Examples of patient-support programs include (1) compliance programs in which consenting patients given a specific medication are contacted to gauge how well they are managing their medication; (2) call centers where patients or caregivers can contact the company to obtain further information on a treatment or a particular disease area; and (3) nurse educator programs, for which the company has hired third-party nurses to directly interact with patients to help them properly administer their medications and/or manage their disease [62].

Patient-support programs should affect all healthcare professionals that are directly or indirectly related to patient care, including doctors, pharmacists, nurses, and caregivers, as well as the patients themselves. PCM should entail systematic use of such programs, recognizing that optimal treatment does not end with writing the correct prescription, but in the implementation of solutions that improve our knowledge of the disease, enable greater patient involvement in realizing therapeutic benefits, and ultimately achieve better outcomes.

### The cost of patient-centered medicine

Cost control and sustainability of healthcare systems are common goals of the governments of all countries. Prioritization of health interventions taking into account their efficiency, and the use of thresholds of cost-effectiveness are controversial topics [63]. For example, assessing the cost-effectiveness of interventions being compared is not within the objectives of the recently established Patient-Centered Outcomes Research Institute [64]. However, if the objective of value-based healthcare systems is to achieve the best health outcomes for patients, *within the economic resources available*[8], it seems logical that an objective of PCM must be to generate the best possible results for the patients, taking into account what the payer can afford. It is also important to understand that, in public health systems, the payer will be the system itself whereas the patients or insurers are the payers in private health systems.

It has been argued that applying cost-effectiveness criteria implies a loss of clinical freedom [65]. However, the doctor plays a fundamental role in the efficient use of resources, and economic aspects are always present, either implicitly or explicitly, in their decisions. If resources are limited, particularly in the context of public health systems based on the principal of equity, it is not ethical to make clinical decisions without taking into account the associated costs. Therefore, the solution lies in choosing *the best options that the health system can offer.*

The efficiency of a medical intervention is not an intrinsic characteristic, but rather depends on how it is being used in clinical practice, including in what subgroup of patients, at what dose, and for how long. An essential responsibility of the doctor is to individualize these interventions in terms of cost-effectiveness, while maximizing the outcomes according to the clinical characteristics and preferences of the patients [66]. The choice of an intervention according to its efficiency should not be considered the end, but just the start of clinical freedom [67].

### Summary

The growing role played by patients within the healthcare system, which could be summarized with the slogan “*nothing about me without me*”; the development of personalized therapies, where “*one size does not fit all*”; the problems associated with EBM, focusing on the evaluation of medical interventions in average patients; and the difficulties in patients obtaining some tangible benefit from the results of clinical research, are just some of the factors that have propelled the recent and rapid development of PCM. The objective of PCM is to improve the health outcomes for individual patients, within the context of everyday clinical practice, taking into account the objectives, preferences, and values of each patient, as well as the available economic resources.

PCM requires a paradigm change in the way medicine is being practiced. We are now moving from a provider-centered model based on the beneficence and authoritarianism of the doctor, to a patient-centered model, based on a principle of autonomy. The patient’s preferences, objectives and values should play a fundamental role in shared decision-making, and the doctor becomes the “enabler who supports the patient to make informed decisions”. The final objective should not be to know what is the best treatment for the average patient, but to improve the health outcomes of individual patients, while ensuring the results of medical research have a real impact on healthcare.

This change in paradigm requires a change in the relationship between doctors and patients, in the ethical principles that control the relationship between them, and in education and medical legislation. It also has important implications for research processes. The development of patient-oriented-research is essential for PCM. It seems logical that the methods underpinning EBM and PCM speak different languages. If EBM is characterized by speaking the language of populations, it should concentrate on the evaluation of health interventions, analysis of aggregated data, variables important to doctors and health regulators, inductive reasoning, the predominance of quantitative methods, the analysis of large sample sizes, and the study of similarities and homogeneity; the methods of PCM should speak the language of individuals by focusing on the evaluation of patients, disaggregation of data, variables important to patients, hypothetical/deductive reasoning, the use of qualitative methodology, analysis of subgroups and individuals, and the analysis of heterogeneity and exceptions [68].

Further developments in information-based technologies can help to close the existing gap between research and clinical practice, without which it would be very difficult to evaluate the effectiveness of healthcare interventions or translate the results of clinical research into tangible benefits for patients. Bringing research and clinical practice closer together will provide advances in the road towards EBM [69], lending greater weight to observations and exceptions, and ultimately ensure that the directionality of translational research is “*from bedside-to-bench*”.

Finally, it must be emphasized that PCM is not incompatible with EBM. A collision between the two must be avoided. Both approaches converge on healthcare from different points of view, and must be considered complementary. If EBM follows the path from the individual patient to the average patient, PCM follows the opposite path, from the average patient to the individual patient. It is not possible to practice PCM that is not based on evidence, nor is it possible to practice EBM at a distance from the individual patient.

## Competing interests

JAS is employed by Eli Lilly & Company.

## Pre-publication history

The pre-publication history for this paper can be accessed here:

http://www.biomedcentral.com/1472-6947/13/6/prepub
